# Back to the future: Why we need enzymology to build a synthetic metabolism of the future

**DOI:** 10.3762/bjoc.15.49

**Published:** 2019-02-26

**Authors:** Tobias J Erb

**Affiliations:** 1 Max-Planck-Institute for Terrestrial Microbiology, Department of Biochemistry & Synthetic Metabolism, Karl-von-Frisch-Str. 10, D-35043 Marburg, Germany; 2 LOEWE Center for Synthetic Microbiology (SYNMIKRO), Marburg, Germany

**Keywords:** enzymes, in vitro biochemistry, metabolic engineering, synthetic biology

## Abstract

Biology is turning from an analytical into a synthetic discipline. This is especially apparent in the field of metabolic engineering, where the concept of synthetic metabolism has been recently developed. Compared to classical metabolic engineering efforts, synthetic metabolism aims at creating novel metabolic networks in a rational fashion from bottom-up. However, while the theoretical design of synthetic metabolic networks has made tremendous progress, the actual realization of such synthetic pathways is still lacking behind. This is mostly because of our limitations in enzyme discovery and engineering to provide the parts required to build synthetic metabolism. Here I discuss the current challenges and limitations in synthetic metabolic engineering and elucidate how modern day enzymology can help to build a synthetic metabolism of the future.

## Introduction

One of the most important and disruptive events in the history of chemistry was its transformation from a purely analytical-descriptive into a synthetic-constructive discipline, which took place more than one hundred years ago [1–2]. Understanding the elemental composition of matter as well as the nature and reactivity of the chemical bond enabled chemists to use their knowledge to create new molecules and materials [3–4]. This development provided humankind with new chemical compounds, such as color dyes, pharmaceuticals, as well as polymers and plastics. Given its transforming nature, it is beyond any doubt that synthetic chemistry has been one of the key enabling technologies of the 20th century, which has virtually changed the world we are living in. Biology is currently at the verge of a similar transition [5]. Over the last decades, our ability to analyze and manipulate living systems has provided the intellectual as well as technological basis to create biological features that are new to nature.

## Review

### Classical metabolic engineering: Exploiting natural metabolic networks

A fundamental feature of living systems is metabolism, which can be defined as the dynamic chemistry that allows life to organize itself in three and four dimensions [6]. The incredible metabolic potential of biology is impressively demonstrated by the more than 2,000 different chemical transformations that can simultaneously take place inside of an *Escherichia coli* cell [7–8], as well as by the more than 200,000 different molecules that have been isolated from different biological systems so far [9]. This diversity has inspired generations of biologists to use living cells as small chemical factories for the production of chemicals.

**Figure 1:**
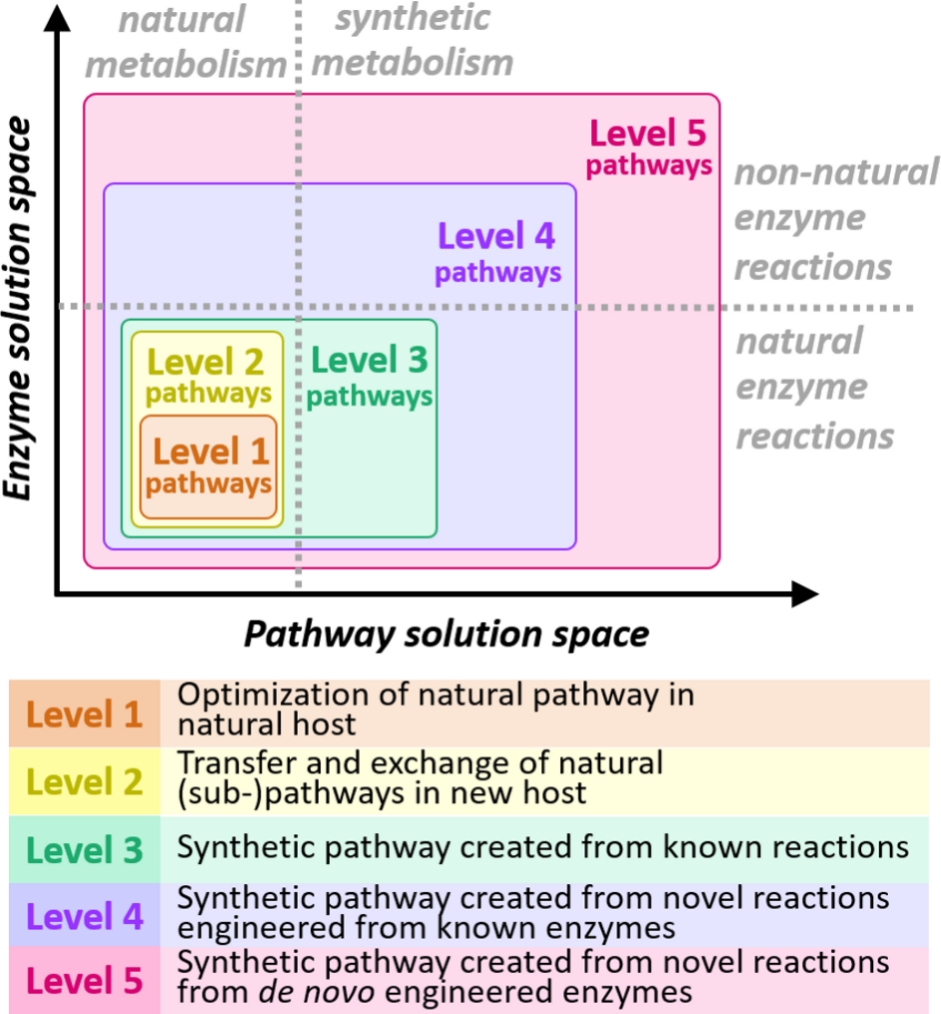
The five levels of metabolic engineering and their definitions according to [11]. The enzyme solution space describes the number of available enzyme reactions. The pathway solution space corresponds to the number of possible pathways that can be constructed. While level 1, 2 and 3 metabolic engineering efforts do not differ in enzyme solution space, because they all rely on known enzyme reactions, level 4 and 5 metabolic engineering efforts are built on new enzyme reactions, which expands the number of pathway solutions.

In the past, many efforts centered on manipulating the metabolism of cells to obtain a target molecule. Most of these approaches were based on the concept of metabolic engineering [10]. According to this concept, known pathways and enzymes are manipulated in such a way that a certain molecule can be produced at high purity and yield from a living bacterial cell [7]. In respect to their complexity (Figure 1), these classical metabolic engineering approaches can be classified as level 1 efforts, i.e., the optimization of a natural pathway in a native organism, or level 2 efforts, i.e., the transplantation or reconstruction of a natural pathway in a new host organism [11].

Classical metabolic engineering efforts, however, are limited in a way that they are still bound to existing pathways and reactions, which limit the accessibility of certain compounds, as well as the efficiency with which those compounds can be produced. In an ideal world, the way a target molecule is produced should not be dictated by the serendipity and constraints of evolution, but be accessible through rational design. However, this requires a fundamental understanding of those principles that are necessary for designing, realizing and operating multi-reaction sequences and metabolic networks de novo.

### Metabolic retrosynthesis: Next level metabolic engineering

Recently, the concept of “synthetic metabolism” was developed that aims at overcoming the limitations provided by natural metabolism through the realization of completely novel metabolic networks [11–12]. The novel networks are designed from first principles based on simple physico-chemical considerations, such as kinetics and thermodynamics. For the design, a starting compound and a target molecule are defined and a short, thermodynamically feasible and energetically efficient route connecting the two molecules is identified. While level 3 engineering efforts aim at creating new pathway solutions by mixing and matching known enzymes from different metabolic pathways, the design efforts in their most advanced form (i.e., level 4 and level 5) do not build on existing enzymes, but only consider plausible chemical transformations and feasible metabolic intermediates [13–16]. In a subsequent realization phase, the corresponding enzymes to realize the theoretical network are identified and/or engineered and a first version of the synthetic network is reconstructed. The network is further optimized or evolved in following rounds in respect to production rate and yield.

**Figure 2:**
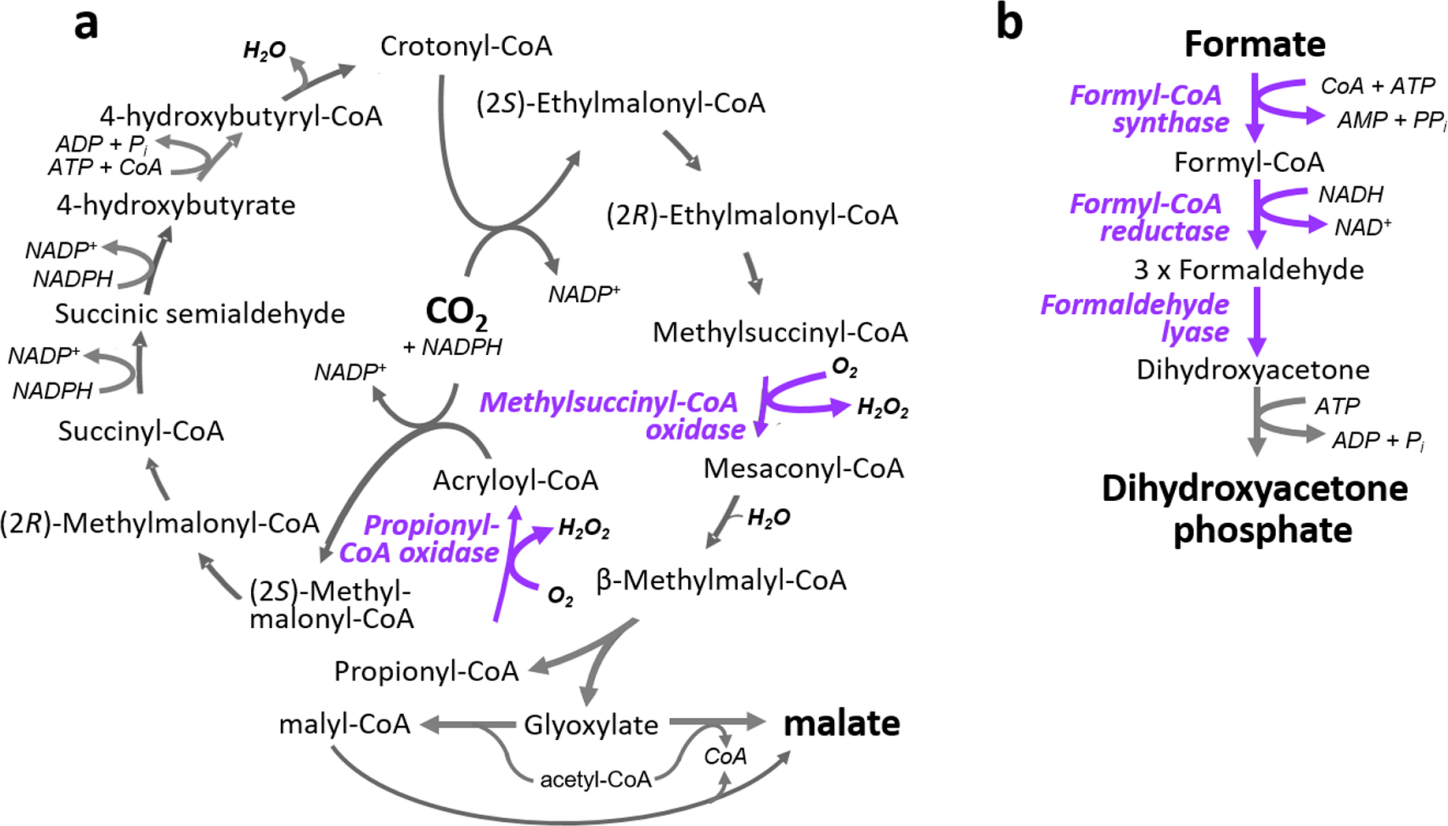
Two level 4 pathways that were recently realized in vitro. (a) The CETCH cycle for CO_2_ fixation [13] and (b) the formolase pathway for formate assimilation [17]. Important enzymes that were engineered to establish these cycles and are mentioned in the text are highlighted in purple.

As an example, several novel level 3 and level 4 pathways for the conversion of CO_2_ into organic acids were developed recently [13–14]. These pathways are predicted to be more efficient than the naturally evolved Calvin cycle of photosynthesis, because they require less energy (ATP, redox power and/or photons) and can be supposedly operated at higher catalytic rates compared to natural carbon fixation. Accordingly, the synthetic CO_2_-fixation cycles should be able to convert more carbon dioxide with less energy in a given time and hence succeed natural photosynthesis in volumetric capacity and energetic efficiency. One of these designs, the so-called CETCH cycle (Figure 2a), a synthetic level 4 pathway for the conversion of CO_2_ into organic acids, was experimentally realized in vitro by combining 17 enzymes (including three engineered ones) from a total of nine different organisms from all three domains of life [13]. Compared to the first version of the cycle, the system was further improved until version 5.4 by almost a factor of 20, indicating that subsequent system optimization might be as important as initial reconstruction [13].

In a similar fashion, multiple level 3 and level 4 routes for the transformation of the one-carbon compound formate into cellular building blocks were designed that should theoretically outcompete natural formate assimilation pathways [18–19]. Some of the level 3 pathways were recently reconstructed in vivo [20–23] and one of the level 4 solutions – the formolase pathway (Figure 2b) – was demonstrated already in vitro [17]. This pathway relies on three new-to nature reactions, the most prominent one being the name-giving formolase reaction, which allows the subsequent condensation of three formaldehyde molecules into the three-carbon compound dihydroxyacetone phosphate [24]. In addition to that, several alternative photorespiration, methanol assimilation, as well as glycolytic pathways of levels 3 and 4 were developed that are supposedly more carbon and energy efficient compared to their naturally evolved equivalents [23,25–28].

Yet, while an increasing number of theoretical designs are proposed, the successful experimental realization of many of these designs in the lab is still falling short. This is especially true for pathways of design levels 4 and 5 that feature novel reactions, for which the corresponding enzymes are unknown (i.e., were not described to date). The realization of these pathways is severely restricted by our limited ability to discover and/or engineer new-to-nature enzymes. Notable exceptions are the formolase pathway and CETCH cycle that required each the establishment of three novel enzymatic reactions for their successful realization. However, other level 4 pathway designs require the establishment of more than ten so-far unknown enzyme reactions, emphasizing the challenge to realize truly synthetic metabolic networks [13].

### The challenge of finding (new) enzymes for synthetic metabolic networks

From above examples it becomes evident that for building completely novel pathways and/or complex reaction cascades, resources are required that provide synthetic biologists with the information to find individual enzymes for a given synthetic metabolic network. More than 116 million proteins were deposited into protein sequence databases, such as UniProtKB [29]. More than 40,000 enzymes were biochemically characterized and the corresponding data is available in specialized enzyme databases, such as BRENDA [30]. This wealth of biological information provides a good starting point to search for enzyme variants that possess a desired catalytic activity.

While existing databases might provide a good resource to find the parts to reconstruct level 3 pathways, this task becomes more challenging in respect to level 4 and level 5 designs that require new-to-nature reactions. How can these new enzyme reactions be identified or established? One option is the de novo-design of enzymes assisted by computational methods, which have been developed over the last couple of years. When combined with experimental evolution and elaborate screening methods, these efforts have allowed to establish completely novel enzyme reactions from scratch [31–34].

However, even though considerable progress has been made in creating enzymes with the help of computational methods [35], it is a complementary (and equally valid) approach to discover and/or engineer novel reactions from the natural diversity of enzymatic scaffolds [36–39]. One example is formaldehyde lyase (or “formolase”) – the key enzyme of the formolase pathway – that was crafted from a benzaldehyde lyase, which showed initially some side reactivity with formaldehyde [17,24]. Other examples are propionyl-CoA oxidase and methylsuccinyl-CoA oxidase in the CETCH cycles that were engineered from a promiscuous short chain acyl-CoA oxidase and a FAD-dependent methylsuccinyl-CoA dehydrogenase, respectively [13,40–41].

These efforts in exploiting the promiscuity of enzymes to create novel catalysts might profit from new computational methods that succeeded in creating active sites of remarkable promiscuous activities in the scaffold of existing enzymes [42]. Such computationally-created “catalytically diverse active sites” could be further developed towards a new activity through directed evolution. Without any question, screening protein sequence and enzyme databases for suitable candidates is key to advance metabolic retrosynthesis. However, there are still some practical issues in extracting the necessary information from different databases. One particular problem of sequence databases like UniProtKB is the high number of misannotated proteins, which is caused by automatized annotation algorithms that are often based on “simple” sequence similarities [43–44]. In selected enzyme (super)families the annotation error can be as high as 90% [45], which masks or even impedes the identification of novel functions within a given enzyme (super)family. An example are reducing enoyl-CoA carboxylases that were for most of the time annotated as ordinary enoyl-CoA reductases, with which they are phylogenetically related [46–47]. Another example are RubisCO-like proteins [48] that are enolases [49], isomerases [50] and transcarboxylases [51], respectively, which are not capable of fixing CO_2_, but are still found very often misannotated as their CO_2_ fixing homologs RubisCO, with which they share a common evolutionary history [52].

A solution to overcome the problem of misannotation might come from novel computational tools that were developed recently to analyze the diversity of enzyme (super)families in respect to new functions [53–55]. While these tools have been successfully used to identify and discover new metabolic pathways (Balskus, etc.), they might as well be used to identify interesting candidate enzymes to be screened for new catalytic reactions in metabolic retrosynthesis. Further improvements in homology modeling and virtual docking are expected to increase accuracy and throughput, which will help to map and predict the substrate and reactions catalyzed by an enzyme superfamily and its individual members in the future.

### Enzyme promiscuity: Key and challenge for synthetic metabolism

Another problem is that even in databases that list the experimentally confirmed activity of enzymes, an important aspect is very often not well documented: substrate (and reaction) promiscuity. Yet, this information is essential to identify suitable candidate templates to engineer or evolve a new activity within the backbone of a given enzyme. For example, although the BRENDA database is probably one of the best resources to learn about the detailed catalytic properties of enzymes, it only provides in selected cases detailed information on the activity of a given enzyme with different substrate analogs. Besides providing the necessary information to identify interesting enzyme candidates for level 4 and level 5 pathway construction, more systematic data on enzyme promiscuity would also allow a more holistic view onto the catalytic (and evolutionary) potential of a complete enzyme superfamily [56].

Note that the information on substrate and/or reaction promiscuity is not only important to establish novel enzyme reactions, it is also of very practical information in the actual construction and optimization of synthetic metabolic networks. One problem in realizing metabolic networks from scratch with enzymes that did not evolve in the same physiological context is that the individual enzymes in such mix-and-match networks are prone to feature side reactivities with substrates or products of other enzymes in the synthetic network, most likely because they lack a common evolutionary history that selects for stringent substrate specificity [57]. These unwanted side reactivities are able to compete with the wanted reactions of the synthetic network and can lead to the accumulation of dead-end products, thus decreasing or even inhibiting flux through the whole synthetic network [58]. Consequently, it is important to learn of such unwanted side reactivities before reconstruction of the network to avoid unfruitful interactions and suboptimal functioning of the system.

Again, the CETCH cycle provides a good example, why information on the promiscuity of enzymes is so important for metabolic retrosynthetic efforts. In the first versions of the synthetic pathway, a promiscuous methylmalyl-CoA lyase caused the accumulation of malyl-CoA from an undesired side reaction of the enzyme with acetyl-CoA, which stalled the cycle. To overcome the problem of unwanted malyl-CoA accumulation, a malyl-CoA thioesterase [59] had to be added to the synthetic network. This enzyme effectively recycles the dead-end metabolite back into two intermediates of the network, malate and free CoA, thus serving as a “proof-reading” enzyme at the periphery of the CETCH cycle to keep the system running. Another problem was posed by the promiscuous activity of propionyl-CoA carboxylase with acetyl-CoA. This problem was solved by replacing the problematic reaction with another enzymatic route. Finally, an initially promiscuous acyl-CoA oxidase was further engineered to increase the catalytic efficiency for the wanted substrate propionyl-CoA compared to the unwanted substrate 4-hydroxybutyryl-CoA by a factor of 50 [13]. Having had known these problematic side reactions beforehand would have probably allowed a more rational design and/or avoided some problems upfront [58].

Yet, it needs to be mentioned that even if complex synthetic metabolic networks can be realized in vitro, this does not mean that these metabolic networks can be easily transplanted into living cells. The introduction of new reactions and metabolites into a host cell is expected to create interactions with the native metabolic and regulatory network of the host. Again, promiscuity poses a major challenge. Even though the metabolites and reactions might be completely non-native to the cell, these intermediates might be still drained due to unwanted side reactions or create unwanted metabolic and regulatory effects that the negatively affect or even prohibit operation of the synthetic metabolic network inside the host. This problem is exacerbated by the fact that for a well-studied organism like E. coli, the function of a large number of enzymes remains still unknown and there are likely to be hundreds if not thousands of unknown reactions and metabolites, often described as catalytic or metabolic “dark matter” [53,60]. Thus, a more detailed understanding of the promiscuity of native enzymes and the interaction of small molecules with the native regulatory network of cells is an important prerequisite to realize synthetic metabolism in the future [61]. In this context, it might also be very interesting to learn, which cellular hosts might be suited best for the transplantation of a given artificial network, or if current approaches to build synthetic cells from the bottom-up might represent a valuable alternative strategy [6].

### Linking enzymology and synthetic biology

In summary, synthetic biology can develop its full potential, if it becomes able to harness the diversity of the millions of different enzyme variants and homologs that naturally exist. While such information is collected and made available by many enzymology and biochemistry laboratories worldwide in a community effort, it is not provided in an optimal way so that it can be used for the synthetic metabolism community. How could this apparent gap be bridged?

First, it will be necessary to collect enzymatic data in a more standardized fashion. As a matter of fact, standardization has been an important driver in the development of synthetic biology. This is probably best demonstrated by the BioBrick System [62] and the multitude of standardized genetic elements that are available for the assembly of complex genetic networks. The STRENDA standard [63–64] might provide a good blue print, how enzyme data could be organized and reported by enzymologists in the future so that the synthetic biologist could better compare and evaluate different enzymes in respect to their suitability for a given pathway.

Second it will be important not only to investigate a given enzyme in respect to its native reaction, but also study its (potential) side reactivities more systematically. For every new enzyme characterized, it would be helpful if the enzymologist tested at least a small set of substrate and/or cofactor analogs. Even though a detailed kinetic data would not necessary be required, the fact that a certain side reactivity exists in the scaffold of a given enzyme would already be a highly useful and relevant information for the synthetic biologist. On the one hand, this information could be used to identify a target enzyme for further engineering to develop the side reactivity as main activity [38–39]. On the other hand, this data would allow the synthetic biologist to anticipate potentially unwanted side reactions in the metabolic network by a given part and take corresponding countermeasures [58].

Third and lastly, there cannot enough enzymes be described. The discovery of new enzymes as well as the characterization of homologs of known enzymes needs to be continued and eventually even intensified. Only these efforts will allow to build an exhaustive library of enzyme parts for level 3, level 4 and level 5 metabolic engineering. At the same time the methods of (re-)engineering and the de-novo design of enzymes need to be further developed. This will allow to further develop and improve catalytic activities in enzymes and create new enzyme reactions that cannot be found naturally. Altogether, these activities will expand the limits of natural metabolism and pave the way for synthetic metabolic networks. Enzymology is far from being an old-fashioned business, its most fruitful era might just have begun.
